# Record of Meteorological Conditions at Buffalo, for August, 1855

**Published:** 1855-10

**Authors:** Sanford B. Hunt


					﻿ART. VIII. — Record of .Meteorological Conditions at Buffalo, for August,
1855. By Sanford B. Hunt, M. D.
— _
tn
S
PS
s
M
PS
•ajnjBjaduiaj,
;o a^uui jsaj-Bojg ci — o	co	ci co	ci	oo	t— t—	t-	rf	oooto w	00	—<
-A'jt I CO 'ijl tJ<	c-	UP CO	to	CO	O CO	00	44	Cl CO Cl	44	d
n	T;	d Cl —I CD — CO	d	—<	CO CO	CO	UP	UP CO O Cl	CO	CO
-pioinjj	uBQjviao t— oo	t—	go t—	oo	oo	go oo	t—	cd	co t- oo t—_________________go_t^
o	‘J^od	up d t- ci 1.0. 00	oo	d.	o? 01	00	00	co 01 co up	co	up
5	-J48Q	rrBaj^j t' r a	o	ct i-'	-?	co	Lt t'	co	'4	06 o co	t—	—■
q ____________CO CO t—	CO	CO UP	CO CO KO	IQ	co	CO	UP UP UP_______________________co_co
•ojojb	. . co	_	co	co.co	t—	co	t— t—	co	co
-xadtnaj, treapt	'f4 o r-‘	ad	o’ ci	-4 cd ci	d’	d	up od t4 -4	•*	ci	00
_______________b- b- b- b- b- to	co co i>-t''»coLQtQco t—
I O 05 O Oi	r-H r-^	cS	r* co	co	o
•	•XirorainTT £r £2 2 £5010	t— 00 ci cc t— up	cm	-04 co 00	up	t-	co
t,	a'psuiuh 00 oo 00 ao ai 00	co c— co 00 00t—	00	co t— co	00	00	00
? __________________________________________________________________________________I
©
E -ltnoj M9H 2 2 °2 S’.	. UP t- rj< . UP CO CD Cl —I co t-	co
_ » . a U 00 co co’d -4 cd’ ci co ci ao co’ up -04 00 up t4	co’	—i
.• tL	COCOt-COOCOCOUPUPCOCOCOCO'^4'04UPUP	co	co
s >,	-----------------------------------------------------------------
• 1-1 -axnjduiaT.......... ................... .	....	.	°Q
X	4 co co co co co co co 00 o up co co d ci oo ci co <-<	t-
	c— t— t— t— co co t—cococot—r- t— up up up co t—_co
,m &S4__________________________________________________________________________£ K.-.£
o £ 3 ,0 0	OQ QQ p «2 cd 02	02	02	? 02
5	_____________—1 -4 —1 —’ O O	co’d ^4 -< i-h’ ci ci d ni 4 4________________________
h spnoiojo^uiy^o 0000 o^oocsoocooco	>
r?	£7 £5 ~	2* 2*^oocQoaoo*-iC5(N'^«-iCQ0>oc'-mot^ m co o co co
?	-iCaiDiuinrr S S S 3	2	~ ° °	° ° H °	°	to —• cr. c*3 -f< —<
4.r.lLULlll ^c-©i>“sOco''tfco^r-r'.{>-aoi>-f^.aoaoiL/3i>’r-r'-r’’OGOC^-co	b-
O __________________________________________________________________
"* T	:
S	• juroj Aiart	~ 1	d ao d. up t-—< —4 . t—co. .	co	ao 00. 00 .	.	co to up d 00 co	d	co cot-	.	Ici
?	’	b	£	22	” £2 22 t- co’ o’ cd	up	—4 —< o o	ci	-4 o co’t- ci up	-4	cd cd up’-4	ci	led
r	.r	_______ -	c—t— t— couosococomcot—	to	co t— t— m	in m co <3 co co co	co	co co to co	up	Ito
^2? ------------------------------------------------------------------------------------
. -ainj.duxa t •	.....................................................................
X	®?£2£2COUPt—t-OCOt-CJOCOCOCOOOOOCOUPdt-OC-COOO co -c CO —l O up
X) GO GO b* H i’X (- ‘O b- x b* b- b- b* to to b* b« i> t- b-	<b-
g’cs• •	. .
=	AA A'A rji 0202’02^ 4s 020202
-----------^oi—i—<-4cicio-tj<cicicici—icicdupoi^4-4r4r4ci___________________
spno[f)jo VnV|co ooooor-ir-c-o'coooaicouoo'^'upiQOi-ii-iCio
r.	2 S it? S ' *2:	t— 4m t-co o co 4-i —1 d ai m ao t~	co
■ •Auprumn ^£J^£^5!£?coo--coco doupci-o<t-ocoupuooup	up
u,	.r. nciaoaocDaooooocpcicoci ooooaot-t—t—ooaocoaocioo	00
© 1 "	——	— ■■ ... - -------------—
. 5 ’jnioj Marr cq co co cd o? up . up up t-co	00—id	coco	d
S h, ’	£ J4i 22 £	<5 £2 c*	cit-4coup’cico’d^4r4-4udod	o’
- ■ .
‘9Jn?4<Iui9J................... ..........................
ocieotooM<oO’-imoin	io
t” ____________h b-to o to	vq ^otrJcot^^otO'^trjcDcoccco
<«	•	.	•	fe	i
r£«o°£ Ax^i x A f A ”. A A A ai	xx
*	O 1*	.	. . .	u4..............................
____________Cd o	—« d o IA	CO CO —I r-1 -< -h —l CO CO d O O d 1-1 O O ’__________
spnoiojoj.iny 000000—<codooooooodoooot-oo
______________—<__________________ —1 ________________—<	—<
! - ------------------------------------------------------ , . .
U -010X83 IO-UTC^P5er5C2'~!'-'^ t- Cl H d CO -0< CO	CO UP Tj< CO	d
S	2 2 2 2 2 2 0 ®	O2’°’ ® <=> ® o o o o o o o’ o o	o
H _____________COCOCOCOCOCOCOCOddCOCOCOCOCOCOCOCOCOCOCOCOCO____________________________co.
o	up o o co	up	co	ci
-uniHJo-ui =j d do S	d	’.	00
’wscrl- ^eo-cfupcot-cocio—idco^upcot—nqcto — ^o-ojgiot^xoo—<’
				

